# Two peculiar new species of *Heteranthera* Ruiz & Pavón (Pontederiaceae) from Brazil, with notes on inflorescence architecture in the family

**DOI:** 10.3897/phytokeys.82.13752

**Published:** 2017-06-28

**Authors:** Marco O. O. Pellegrini, Charles N. Horn

**Affiliations:** 1 Universidade de São Paulo, Departamento de Botânica, Rua do Matão 277, CEP 05508-900, São Paulo, SP, Brazil; 2 Jardim Botânico do Rio de Janeiro, Rua Pacheco Leão 915, CEP 22460-030, Rio de Janeiro, RJ, Brazil; 3 Smithsonian Institution, NMNH, Department of Botany, MRC 166, P.O. Box 37012, Washington DC 20013-7012, USA (current address); 4 Newberry College, Department of Sciences and Mathematics, 2100 College Street, Newberry, SC 29108, USA

**Keywords:** Atlantic Forest, aquatic flora, Commelinales, mudplantains, Neotropical flora, pickerelweed

## Abstract

Two new and peculiar species of *Heteranthera* are herein described. *Heteranthera catharinensis* is unique in the genus due to its glomerulate, many-flowered inflorescences, in which the flowers are restricted to the base and apex of the cincinni. It also possesses the biggest flowers in the *H. reniformis* Ruiz & Pavón species complex, with glabrous perianth lobes, medial filament, and style. On the other hand, *Heteranthera pumila* is described as the smallest known species of Pontederiaceae, with its dwarf stature, petiolate leaves with especially diminute blades, inflorescences 1–2–(3)-flowered, peduncle densely covered with glandular hairs, basal bract with glandular hairs at base, and smooth seeds, rarely possessing 7–9 inconspicuous longitudinal wings. We present detailed descriptions, illustrations, comments, a distribution map, conservation assessments for the new species, and an identification key to the Brazilian species of *Heteranthera* *s.l.* Finally, we discuss inflorescence morphology and terminology in Pontederiaceae, characterizing it as thyrsoid.

## Introduction

*Heteranthera* Ruiz & Pavón, *nom. cons.* is currently the largest genus of Pontederiaceae, comprising 12 neotropical species, and two paleotropical species restricted to continental Africa and Madagascar [i.e. *H. callifolia* Rchb. *ex* Kunth, and *H. lutea* (H.Perrier) M.Pell.] (Horn 1985; Pellegrini 2017). In Brazil, *Heteranthera* is currently represented by nine species (i.e. 75% of the diversity of the genus), widely distributed throughout permanent and temporary freshwater bodies in the country (BFG 2015). The genus is especially diverse in the Atlantic Forest domain, where seven species are known to occur (BFG 2015).

*Heteranthera* was described based on Peruvian collections of *H. reniformis* Ruiz & Pavón, being originally characterized by its three dimorphic stamens, six-lobed perianth, and polyspermic capsules (Ruiz López and Pavón 1794). Since then, several different genera have been segregated or described to accommodate species which were considered aberrant from *Heteranthera* *s.s.* (i.e. *Eurystemon* Alexander, *Hydrothrix* Hook.f., *Schollera* Schreb., *nom. illeg.*, *Scholleropsis* H.Perrier, and *Zosterella* Small). These genera were described mainly based on autapomorphic characters, such as vegetative differences (e.g. number of projections in the ligule, misinterpreted as verticillate leaves) and minor reproductive characters (e.g. number of flowers per inflorescence, number of fertile stamens, filament inflation, and anther curvature at post-anthesis; Pellegrini 2017). Several phylogenetic studies evidenced the paraphyly of *Heteranthera* (Eckenwalder and Barrett 1986; Graham and Barrett 1995; Kohn et al. 1996; Barrett and Graham 1997; Graham et al. 1998; Ness et al. 2011), and pointed towards a broader sense of the genus, which was subsequently accepted in taxonomic and floristic treatments worldwide (Horn 1987a, 1987b, 2002; Horn and Haynes 2001; BFG 2015; Pellegrini 2017). The genus is currently easily recognized by its non-pulvinate petiolate leaves, inflorescence reduced to a solitary cincinnus, stamens (1–)3, staminodes sometimes present, the lack of septal nectaries, and its unevenly trilobate stigma (Pellegrini 2017; Pellegrini and Horn, unpublished data).

Despite *Heteranthera* being currently monophyletic and well circumscribed (Pellegrini 2017), some widely distributed taxa are still problematic. The main neotropical species complex is represented by *H. reniformis* *s.l.*, which also includes the *H. multiflora* *s.l.* subcomplex. *Heteranthera reniformis* *s.l.* is the most widespread and morphologically variable taxon in the genus (Horn 1985). It is also known to be an aggressive weed, especially in rice fields around the world (Ferrero 1996; Vescovi et al. 1996; SWSS 1998; Karov et al. 2005; Domingos et al. 2005; Arakaki 2013; Csurhes 2016). Nonetheless, species identification is extremely difficult due to the poorly understood specific limits in this group. As part of our ongoing systematic studies in Pontederiaceae, based on extensive field and herbaria studies, we describe two peculiar new species segregated from *H. reniformis*, and clarify the complex’s composition and morphological characterization.

## Methods

The description and phenology of the species is based on herbaria, spirit, and fresh material, and is complemented by literature information. Specimens from the following herbaria were also analyzed: AAU, ALCB, B, BA, BAF, BHCB, BHZB, BM, BOL, BOTU, BR, C, CAS, CEPEC, CESJ, COL, CORD, CTES, CVRD, E, ESA, F, FCAB, FLOR, FUEL, FURB, G, GH, GUA, HAMAB, HAS, HB, HBR, HERBAM, HRB, HRCB, HSTM, HUEFS, HUFSJ, HURB, IAC, ICN, INPA, IPA, K, KANU, LIL, LP, MA, MBM, MBML, MG, MO, MVM, MY, NBYC, NY, PMSP, PRC, R, RB, RFA, RFFP, S, SJRP, SP, SPF, UEC, UNA, UPCB, and US (herbaria acronyms according to Thiers, continuously updated). The distribution of the species is based on herbaria materials, field data, and literature. The classification of the vegetation patterns follows IBGE (2012). The indumenta and shapes terminology follows Radford et al. (1974); the inflorescence terminology and morphology follows Weberling (1965, 1989) and Panigo et al. (2011); the fruit terminology follows Spjut (1994); and general terminology follows Horn (1985). The conservation status is proposed following the recommendations of IUCN Red List Categories and Criteria, Version 3.1 (IUCN 2001). GeoCAT (Bachman et al. 2011) was used for calculating the Extent of Occurrence (EOO) and the Area of Occurrence (AOO).

## Results

We update the number of species of *Heteranthera* in Brazil from nine to 11, including the number of species endemic to the country from one to three, and the total number of species in the genus from 14 to 16. Both new species belong to the *H. reniformis* species complex, being differentiated from *H. reniformis* *s.s.* based on several reproductive features (Table 1). We provide detailed morphological descriptions, comments, illustrations, and a distribution map for the new species, along with an identification key for the species of *Heteranthera* in Brazil. A morphological characterization and general comments are also provided for the *H. reniformis* species complex, with special attention to *H. multiflora* (Griseb.) C.N.Horn.

### Taxonomy

#### 1. Heteranthera catharinensis

Taxon classificationPlantaeORDOFAMILIA

C.N.Horn & M.Pell. sp. nov.

urn:lsid:ipni.org:names:77163813-1

Figs 1 , 2 , 3

##### Diagnosis.

Similar to *Heteranthera reniformis* Ruiz & Pavón due to is petiolate leaves with reniform to broadly cordate blades, glandular-pubescent cincinnus axis, perianth lobes with a 5+1 arrangement, and straight filaments. It is unique due to its 3.2–5.5 cm long, glabrous peduncles, basal bract with spatulate-mucronate apex, 6–17-flowered, glomerulate cincinnus; externally glabrous perianth lobes, central superior perianth lobe 6.6–9.2 mm long, central stamen with glabrous filament, lateral anthers 1–1.8 mm long, central anther 1.7–2.4 mm long, and glabrous style.

**Table 1.** Morphological characters differentiating the South American species of *Heteranthera reniformis* species complex. States in bold represent unique or distinguishing characteristics for that species. ^*^Populations of *H. multiflora* in Argentina and Paraguay have a much more elongate cincinnus with only a few flowers within the basal bract (spathe). ^**^In North America, *H. multiflora* has smaller perianth tube lengths.

Characters	*H. catharinensis*	*H. multiflora*	*H. pumila*	*H. reniformis*
**Leaf blade width**	(14–)30–46 mm	29–65 mm	**3.2–12.1 mm**	13–40 mm
**Peduncle**	**3.2–5.5 cm long**, glabrous	0.1–1.2 cm long, glabrous	0.5–3.4 cm long, **glandular-pubescent**	0.5–2.2(–3) cm long, glabrous
**Basal bract (spathe)**	**Spatulate-mucronate**	Mucronate	**Aristate**	Mucronate
**Flower arrangement**	**Glomerulate (condensed at the base and apex of the cincinnus)**	Evenly distributed along the cincinnus	Evenly distributed along the cincinnus	Evenly distributed along the cincinnus
**Cincinnus**	6–17-flowered, main axis glandular-pubescent	3–13 flowered, **main axis glabrous**	**1–2(–3)-flowered**, main axis glandular-pubescent	3–8-flowered, main axis glandular-pubescent
**Flowers exerted from the basal bract (spathe)**	**5–15**	0–3(–10) ^*^	0(–1)	0–3
**Perianth tube length**	5–7.5 mm	(3–)6–10 mm ^**^	4.9–7.3 mm	**2.5–5 mm**
**Perianth lobes pubescence**	**Glabrous**	Glandular-pubescent	Glandular-pubescent	Glandular-pubescent
**Central superior perianth lobe length**	**6.6–9.2 mm**	3–7.5 mm	3.6–4 mm	2.3–5 mm
**Lateral stamens**	Filaments barbate with hairs of unknown color, **anthers 1.0–1.8 mm long**	Filaments barbate with purple hairs, anthers 0.5–1.1 mm long	Filaments barbate with lilac to pink hairs, anthers 0.4–0.6 mm long	**Filaments barbate with white hairs**, anthers 0.2–0.6 mm long
**Central stamen**	**Filament glabrous, anther 1.7–2.4 mm long**	Filament barbate with purple hairs, anther 1–1.9 mm long	Filament villose with white hairs, anther 1.2–1.6 mm long	Filament villose with white hairs, anther 0.6–1.4 mm long
**Seeds**	Unknown	Testa with 9–12 conspicuous longitudinal wings	**Testa smooth or with 7–9 inconspicuous longitudinal wings**	Testa with 8–14 conspicuous longitudinal wings

##### Type.

**BRAZIL. Santa Catarina**: Ipumirim, 4–7 km south of the Rio Irani, 26°59'S, 52°11'W, alt. 500–600 m, 9 Dec 1964, L.B. Smith & R.M. Klein 13919 (holotype: US barcode US01936706!; isotypes: FLOR barcode FLOR3365!, LP!, MO!, NY!, R!).

**Figure 1.**
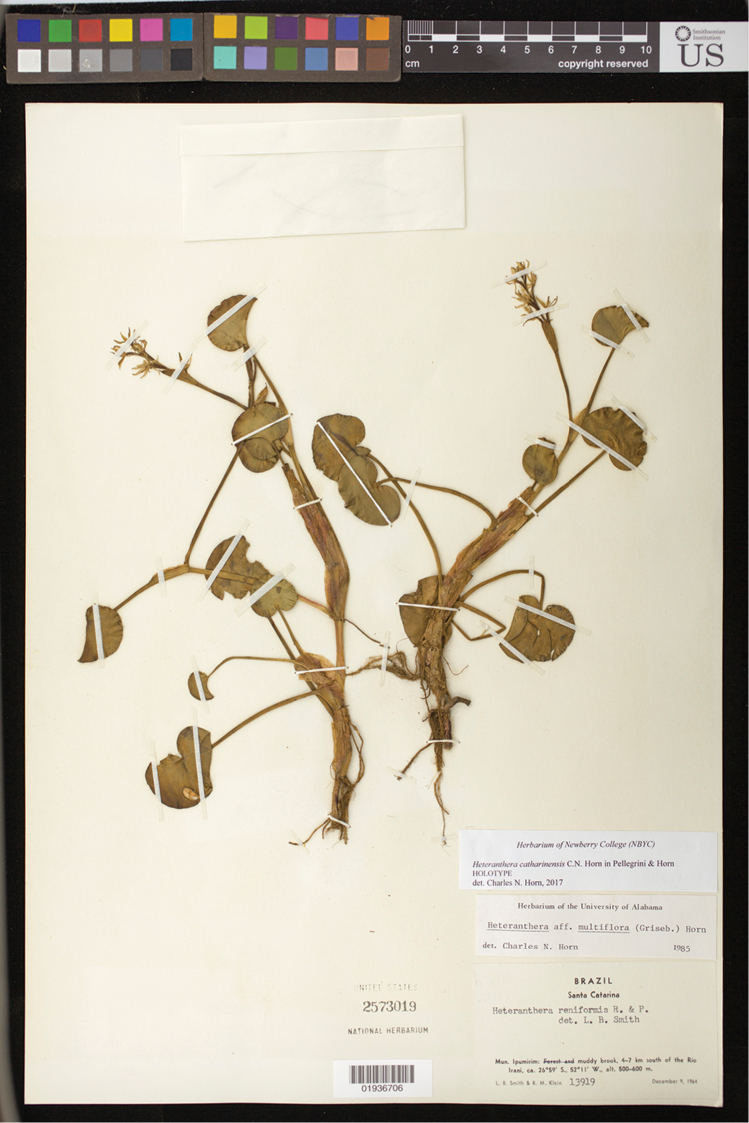
Holotype of *Heteranthera catharinensis* C.N.Horn & M.Pell. Image courtesy of the Smithsonian Institution, NMNH, US herbarium.

**Figure 2.**
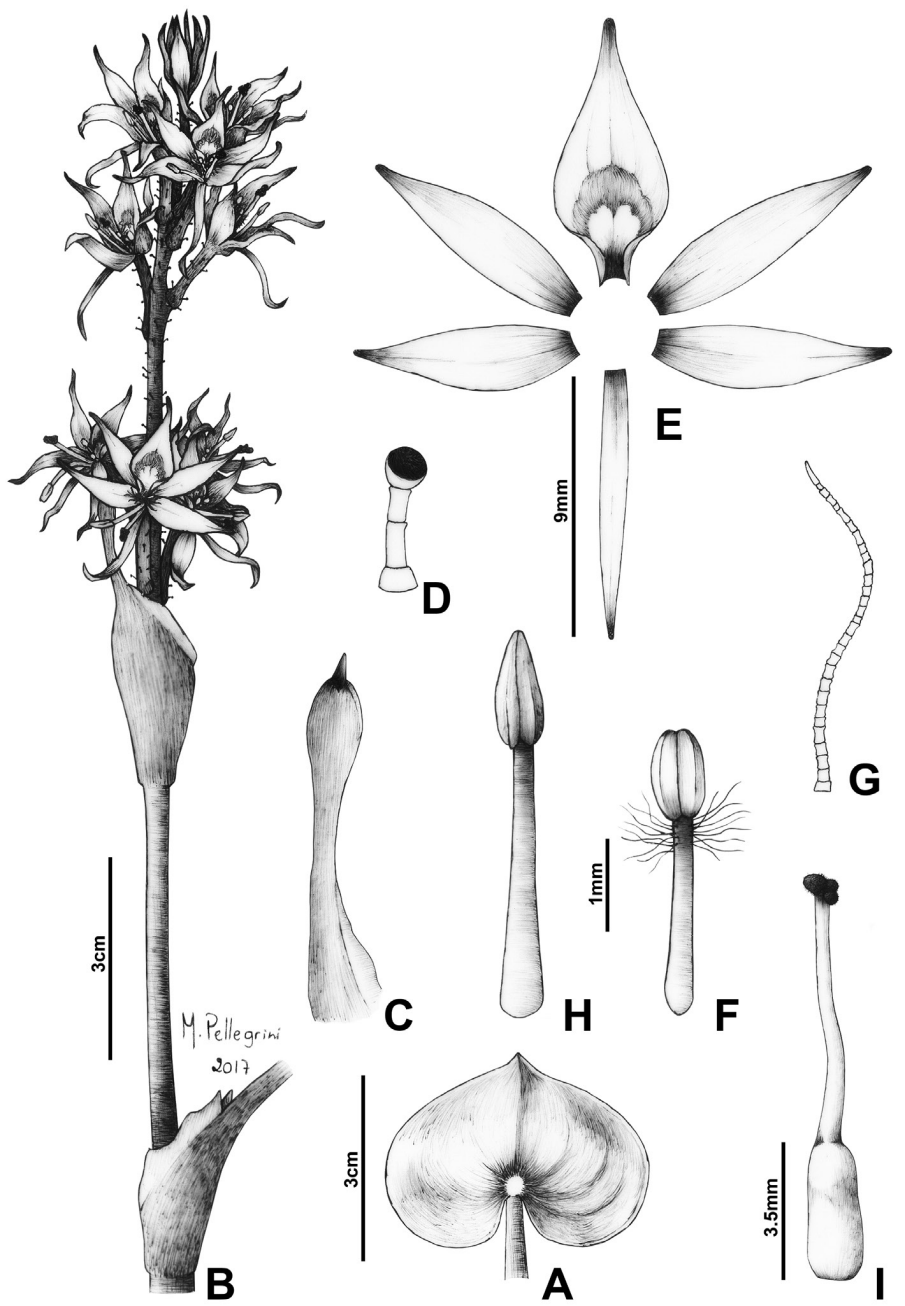
Line drawing of *Heteranthera catharinensis* C.N.Horn & M.Pell. **A** Detail of the leaf blade **B** Detail of the apex of the stem, showing the ligule and an inflorescence at anthesis **C** Detail of the basal bract, showing the spatulate-mucronate apex **D** Glandular hair from the cincinnus axis and floral tube **E** Dissected perianth lobes, showing the 5+1 arrangement **F** Lateral stamen **G** Uniseriate hair from the lateral stamen **H** Central stamen **I** Gynoecium, showing the glabrous style and unevenly trilobate stigma. Illustration by M.O.O. Pellegrini, based on the paratype (Smith & Reitz 9103, US).

##### Description.

*Herbs* annual or short-lived perennials. *Roots* thin, delicate, unbranched, white. *Stems* repent on the substrate or floating in shallow water, delicate, spongy, rooting at the nodes; internodes 1.6–4.3 cm long, glabrous. *leaves* not seen. *Petiolate leaves* distichously-alternate, distributed along the stem, floating to emergent; sheaths 2.6–5.5 cm long, glabrous, covered with mucilage, longitudinally split and green when mature, ligule 2-parted, barely surpassing the sheath, 0.1–0.3 mm long, membranous, light green, glabrous, apex triangular; petiole 3.3–21 cm long, not inflated, glabrous; blades 1.3–3.3 × (1.4–)3–4.6 cm, reniform to broadly cordate, membranous, glabrous, base cordate, margins glabrous, apex obtuse to slightly acute. *Inflorescences* axillary or apparently terminal, reduced to a solitary pedunculate cincinnus; peduncle 3.2–5.5 cm long, glabrous; basal bract (spathe) 1.6–3.3 × 0.3–0.5 cm, spathaceous, elliptic, conduplicate, glabrous, green, margins hyaline, apex spatulate-mucronate; cincinnus bract absent; cincinnus 6–17-flowered, flowers congested at the base and apex of the cincinnus, 1–2 flowers included in the basal bract, axis 3–6.5 cm long, slightly to densely glandular-pubescent. *Flowers* bisexual, tubular, chasmogamous, sessile, enantiostylous; floral buds narrowly ellipsoid, light green, glabrous; perianth tube 5–7.5 mm long, light green, glandular-pubescent, lobes 5 superior and 1 inferior, white, lateral superior lobes 6.6–8.3 × 1.2–2.5 mm, elliptic, base cuneate, apex acute to acuminate, central superior lobe 6.6–9.2 × 1.6–2.5 mm, ovate to broadly ovate, base obtuse, slightly involute, apex acute, with a nectar guide at base, pale to medium yellowish green with an upper mauve to vinaceous spot, inferior lobe 6.5–9.5 × 0.4–1 mm, linear elliptic, base cuneate, apex acuminate; stamens 3, lateral stamens with filaments straight, 1.5–2 mm long, not inflated, apically barbate with eglandular, multi-celled hairs, anthers 1–1.8 × 0.3–0.4 mm, oblongoid to ellipsoid, yellow, central stamen with filament straight, 3–3.6 mm long, not inflated, glabrous, anthers 1.7–2.4 × 0.4–0.6 mm, ovate to slightly sagittate, white; ovary 3.2–3.8 × 1.1–1.3 mm, linear ovoid to linear oblongoid, glabrous, 1-locular, placentation intrusive-parietal, style gently sigmoid, 5.1–9.3 mm long, glabrous, stigma unevenly trilobate, densely glandular-pubescent. *Capsules* not seen; persistent perianth base (anthocarp) smooth, medium brown. *Seeds* not seen.

##### Specimens seen

**(paratypes). BRAZIL. Santa Catarina**: Caçador, slough, 33 km W of Caçador, fl., 23 Dec 1956, L.B. Smith & R. Reitz 9103 (HBR!, NY!, P barcode P02188433!, US barcode US01936705!)

##### Etymology.

The epithet makes reference to the type locality, the state of Santa Catarina, Brazil.

##### Distribution, habitat and ecology.

**Figure 3.**
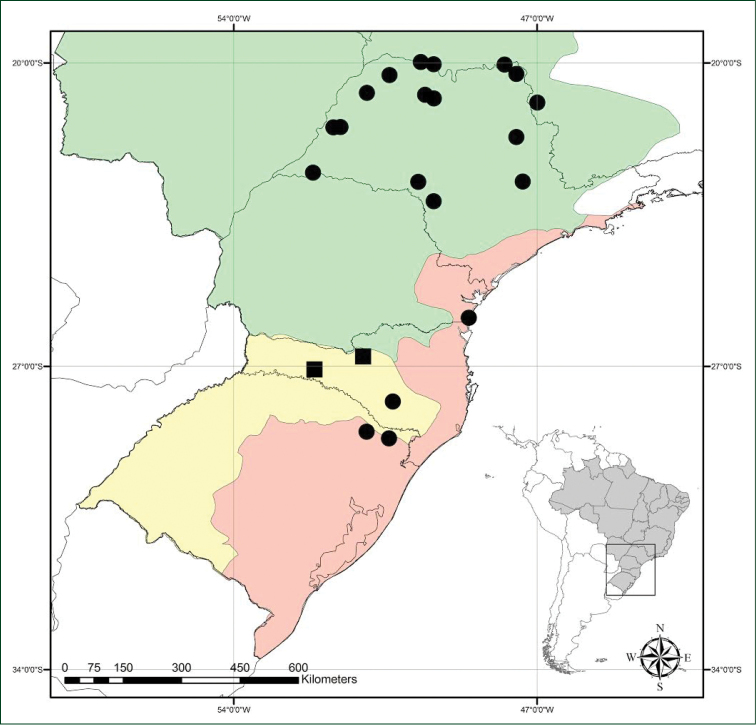
Distribution map. ■ *Heteranthera catharinensis* C.N.Horn & M.Pell. ● *H. pumila* M.Pell. & C.N.Horn. Green– Paraná watershed; Yellow– Uruguay watershed; Red– Southeastern Atlantic watershed; following ANA – Agência Nacional de Águas (2002).

*Heteranthera catharinensis* is currently endemic to the state of Santa Catarina, in the Atlantic Forest domain (Fig. 3). Is was found growing on open marshy areas and slow water environments within the Uruguay River watershed.

##### Phenology.

*Heteranthera catharinensis* can be found in bloom in December. Unfortunately, neither of the two currently known collections present mature fruits, thus fruiting time remains unknown.

##### Conservation status.

Following the IUCN recommendations (IUCN 2001), *H. catharinensis* should be considered as Data Deficient (DD), since it is known from only two collections, which are more than 50 years old.

##### Morphological notes.

The inflorescence of *H. catharinensis* is extremely peculiar, meriting explanation. The glomerulate appearance of the inflorescence (i.e. flowers congested at the base and apex of the inflorescence) seems to be due to changes in the length of the cincinnus internodes. The first one to three internodes are contracted, similarly to most species in the genus, thus making the basalmost flowers to be partially enclosed by the basal bract. Nonetheless, the following internode is considerably and consistently elongated, being commonly three to five times longer than the previous internodes. The subsequent internodes are also contracted, giving the impression that the flowers are also congested at the apex of the inflorescence. This alternation between contracted and elongated internodes, produces a unique inflorescence architecture in the genus (Fig. 2B).

##### Affinities.

*Heteranthera catharinensis* is morphologically similar to *H. reniformis* *s.s.* due to its petiolate leaves with reniform to broadly cordate blades, pedunculate inflorescences, cincinnus axis glandular-pubescent, glandular-pubescent perianth tube, perianth lobes with a 5+1 arrangement and acute to acuminate at apex, lateral stamens apically barbate, and intrusive-parietal placentation (Horn 1985). It is also superficially similar to *H. multiflora* *s.l.* due to its bigger stature, many-flowered inflorescence with few flowers included in the basal bract, and gross floral morphology (Horn 1985). Nonetheless, *H. catharinensis* can be easily differentiated from all remaining species of *Heteranthera* by its unique inflorescence architecture (where flowers are congested at the base and the apex of the cincinnus), larger flowers size, numerous flowers on an elongate axis, main axis many times longer that the basal bract, and basal bract with spatulate-mucronate apex. Aside from that, specimens of *H. catharinensis* have been erroneously identified as *H. peduncularis* Benth, due to their robust habit and long inflorescences. However, both species can be easily differentiated based on inflorescence architecture, and pubescence of the tepals and filaments. Furthermore, *H. catharinensis* has larger floral features, when compared to the remaining species of the *H. reniformis* complex, including longer perianth lobes and larger anthers. It is also the only species in the complex with externally glabrous perianth lobes, and glabrous central filament and style (Table 1).

#### 2. Heteranthera pumila

Taxon classificationPlantaeORDOFAMILIA

M.Pell. & C.N.Horn sp. nov.

urn:lsid:ipni.org:names:77163814-1

Figs 3 , 4 , 5

##### Diagnosis.

Similar to *H. reniformis* Ruiz & Pavón due to its petiolate leaves with blades two or more times wider than long, reniform to broadly cordate, cincinnus enclosed by the basal bract, glandular-pubescent cincinnus axis, perianth lobes with a 5+1 arrangement and acute to acuminate at apex, filaments straight, and intrusive-parietal placentation. It differs due to its diminute petiolate leaves [3.5–11.8–(13.2) × 3.2–12.1 mm], inflorescences 1–2–(3)-flowered, peduncle densely glandular-pubescent, basal bract glandular-pubescent at base, apex aristate, flowers pale lilac to lilac or light pink, seeds smooth or with 7–9 inconspicuous longitudinal wings.

##### Type.

BRAZIL. São Paulo: Piraju, várzea do rio Paranapanema, na divisa com o município de Manduri, 23°07'50"S 49°19'32"W, fl., fr., 10 Oct 2016, M.O.O. Pellegrini & R.F. Almeida 495 (holotype: RB!; isotypes: NBYC!, SPF!, US!).

**Figure 4.**
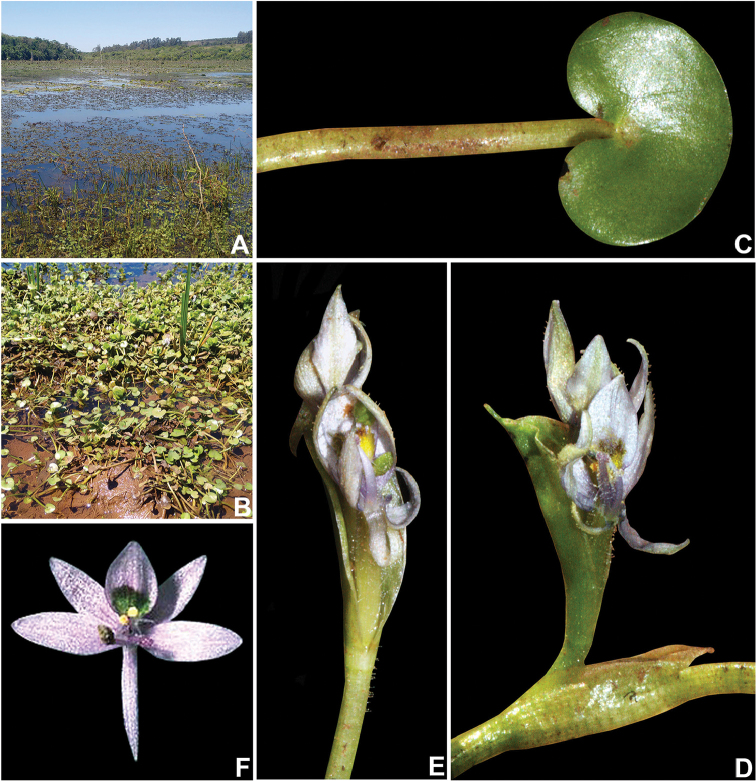
Field photos of *Heteranthera pumila* M.Pell. & C.N.Horn. **A** Bog at the Paranapanema river, Piraju, São Paulo, Brazil **B** Habit, showing the dense subpopulation at the muddy shore of the bog **C** Leaf **D** Detail of the apex of the stem, showing the ligule and the inflorescence **E** Detail of the inflorescence, showing the glandular hairs at the peduncle, base of the basal bract and cincinnus **F** Front view of the flower, the shape of the perianth lobes and the color of the nectar guide. Photographs A–E by M.O.O. Pellegrini, F by V. Bittrich.

##### Description.

*Herbs* annual or short-lived perennials. *Roots* thin, delicate, unbranched, white. *Stems* repent on the substrate or floating in shallow water, delicate, spongy, rooting at the nodes; internodes 1.7–64.1 mm long, glabrous. *leaves* not seen. *Petiolate leaves* distichously-alternate, distributed along the stem, floating to emergent; sheaths 2.8–7.5 mm long, glabrous, covered with mucilage, longitudinally split and light green when mature, ligule 2-parted, surpassing the sheath, 0.2–0.8 mm long, membranous, light green, glabrous, apex triangular; petiole 8.5–82.9 mm long, not inflated, glabrous; blades 3.5–11.8–(13.2) × 3.2–12.1 mm, cordate to broadly cordate to reniform, rarely narrowly cordate, membranous, glabrous, base cordate, margins glabrous, apex acute to obtuse. *Inflorescences* axillary or apparently terminal, reduced to a solitary pedunculate cincinnus; peduncle 0.5–3.4 cm long, deflexed and submerged in fruit, densely glandular-pubescent; basal bract (spathe) 0.9–1.9 × 0.4–0.8 cm, spathaceous, broadly elliptic, conduplicate, green, glandular-pubescent at base, margins hyaline, apex aristate; cincinnus bract absent; cincinnus 1–2–(3)-flowered, all flowers included in the basal bract, when present the third flower always exerted, axis 0.2–1.8 mm long, densely glandular-pubescent. *Flowers* bisexual, tubular, chasmogamous, sessile, enantiostylous; floral buds narrowly ovoid, light green to lilac or pink, densely glandular-pubescent; perianth tube 4.9–7.3 mm long, light green, densely covered with glandular hairs, lobes 5 superior and 1 inferior, pale lilac to lilac or light pink, lateral superior lobes 3.6–5 × 0.8–1.4 mm, elliptic, base cuneate, apex acute to acuminate, central superior lobe 3.6–4 × 1.7–2.1 mm, ovate to broadly ovate, base obtuse to rounded, slightly involute, apex acute, with a nectar guide at base, yellowish green to green with an upper vinaceous to brown spot, inferior lobe 4.2–4.9 × 0.5–0.8 mm, narrowly elliptic to linear elliptic, base cuneate, apex acuminate; stamens 3, lateral stamens with filaments straight, 1.6–1.8 mm long, pale lilac to light pink, not inflated, apically barbate with eglandular, multi-celled, lilac to pink hairs, anthers 0.4–0.6 × 0.3–0.5 mm, broadly oblongoid to quadrangular, yellow, central stamen with filament straight, 2–2.3 mm long, lilac to pink, not inflated, medially sparsely villose with eglandular, white hairs, anthers 1.2–1.6 × 0.3–0.5 mm, ellipsoid, greyish blue to greyish mauve; ovary 3.1–3.5 × 1–1.2 mm, linear ovoid, glabrous, 1-locular, placentation intrusive-parietal, style gently sigmoid, 4.2–5.1 mm long, lilac to pink, terete, densely villose in the distal portion with eglandular, white hairs, stigma unevenly trilobate, purple to pink, densely glandular-pubescent. *Capsule* 5.3–7.2 × 1.1–1.9 mm, linear ovoid, glabrous, smooth, light green when immature, light to medium brown when mature; persistent perianth base (anthocarp) smooth, medium to dark brown. *Seeds* 0.5–0.7 × 0.2–0.3 mm, oblongoid, light brown to yellowish brown, testa smooth, sometimes with 7–9 inconspicuous longitudinal wings; hilum punctate; embryotega dorsal, inconspicuous, without a prominent apicule.

##### Specimens seen

**(paratypes). BRAZIL. Minas Gerais**: São Sebastião do Paraíso, Fazenda Fortaleza, fl., 20 Apr 1945, A.C. Brade & A. Barbosa 17846 (RB, SP, UNA). **Paraná**: Guaratuba, Boa Vista, fl., 28 Jan 1964, G. Hatschbach 11078 (MBM); Rio da Divisa, fl., fr., 16 Dec 1971, G. Hatschbach 28523 (MBM, UPCB). **Rio Grande do Sul**: Bom Jesus, Rio Socorro, fl., 19 Feb 2008, Grupo de Estudos Reófitas UHBG 2116 (MBM). Vacaria, vale do Rio Ibitíria, ca. 30 km NE de Vacaria, fl., s.dat., J.C. Lindeman et al. s.n. (ICN9466). **Santa Catarina**: Lages, Santo Antônio, near Passo de Socorro, estrada de rodagem Federal km 67–71, south of Lages, fl., 14 Jan 1957, L.B. Smith & R. Reitz 9959 (HBR, RFA, US). **São Paulo**: Americana, Praia Azul, fl., 2 Mar 1993, Faria 96/16 (UEC). Bálsamo, estrada sentido Bálsamo-Mirassolândia, fl., 30 Jan 1997, A.D. Faria et al. 97/350 (UEC). Dracena, margem do Rio do Peixe, fl., fr., 7 Sep 1995, L.C. Bernacci et al. 2124 (IAC, SP, SPF, UEC). Estrela D’Oeste, SP-320, lago localizado na Fazenda Santo Antônio, lado direito da pista no sentido Estrela D’Oeste-Jales, fl., fr., 30 Jan 1997, L.Y.S. Aona et al. 97/167 (UEC). Igarapava, lagoa localizada na Fazenda Flor das Frutas, lado direito da pista no sentido Igarapava-Rifaina, na altura do km 16, fl., 15 Jan 1997, A.D. Faria et al. 97/102 (UEC). Ouro Verde, SP-563, km 113, Ponte Nova, Rio do Peixe, fl., 10 Jul 1996, A.D. Faria et al. 96/122 (UEC); loc. cit., fl., fr., 10 Jul 1996, A.D. Faria et al. 96/130 (BOTU, IAC, SP, SPF, UEC). Paulo de Faria, fl., Oct 1994, V.C. Souza et al. 12294 (ESA, IAC, UEC). Pedregulho, rodovia Antônio Giolo, acesso à Estreito, solo encharcado próximo à uma cachoeira, fl., fr., 14 Jan 1997, A.D. Faria et al. 97/64 (UEC). Piraju, várzea do Ribeirão São Bartolomeu, fl., fr., 15 May 1996, E.L.M. Catharino et al. 2090 (PMSP). Riolândia, brejo localizado em estrada de terra no sentido Riolândia-Paulo de Faria, fl., 29 Jan 1997, L.Y.S. Aona et al. 97/152 (UEC). Santa Rita do Passa Quatro, rodovia Anhanguera, km 239, Sítio Aubiri, fl., 13 Jan 1997, A.D. Faria et al. 97/20 (UEC). São José do Rio Preto, represa, fl., 25 Nov 1965, G. Marinis & E.M.P. Martins 20 (FUEL, SJRP, SP); Estação Experimental de Zootecnia de São José do Rio Preto, fl., 28 Dec 1977, M.A. Coleman 220 (SP). São Pedro do Turvo, 8 km da estrada em direção à Marília, desvio em estrada de terra ca. 3.5 km, 49°70'W 22°48'S, est., 9 Dec 1994, M.C.E. Amaral & V. Bittrich 94/48 (UEC). Sud Mennucci, distrito de Bandeirantes D’Oeste, fl., 4 Aug 1995, M.R. Pereira-Noronha et al. 1552 (SP). Teodoro Sampaio, margem do lago ao lado da estrada Teodoro Sampaio-Planalto, ca. Km 11.5, fl., Oct 1997, L.Y.S. Aona et al. 97/241 (UEC).

##### Etymology.

The epithet means “small”, making allusion to the small stature of the new species, especially its diminute leaf blades.

##### Distribution, habitat and ecology.

*Heteranthera pumila* is endemic to the Paraná, Uruguay, and Southeastern Atlantic watersheds, in the Atlantic Forest domain. It is restricted to Brazil, in the states of Minas Gerais, São Paulo, Paraná, Santa Catarina and Rio Grande do Sul (Fig. 2), growing on open marshy areas and slow water environments along the Paraná, Paranapanema and Rio Grande rivers (and their respective tributaries), from 700 to 1,800 meters above the sea level. It is very likely that *H. pumila* also reaches the state of Mato Grosso do Sul. Nonetheless, we have been unable, so far, to find any vouchers from this state in the visited herbaria.

##### Phenology.

*Heteranthera pumila* blooms throughout the year, with flowering peaks during the wet season, and was found in fruit from September to October and from January to March.

##### Conservation status.

*Heteranthera pumila* is widely distributed across the upper Paraná, Uruguay, and Southeastern Atlantic watersheds, with a wide EOO (ca. 318,815.754 km^2^) which would render this species as Least Concern. On the other hand, its AOO is considerably smaller (ca. 88.000 km^2^), which would render *H. pumila* as Endangered. The Paraná, Uruguay, and Southeastern Atlantic watersheds cover eight Brazilian states (Distrito Federal, Goiás, Mato Grosso do Sul, Minas Gerais, Rio Grande do Sul, Santa Catarina, São Paulo, and Paraná), embedded in the Atlantic Forest and Cerrado domains. Its main tributaries are the Iguaçu, Paranaíba, Paranapanema, Rio Grande and Tietê rivers. It possesses the greatest energy generation potential in Brazil, with 176 active hydropower plants, the largest being Itaipu, Furnas, Porto Primavera and Marimbondo. Nonetheless, all the major rivers are currently saturated with hydropower plants, and new projects aim to occupy the smaller tributaries, in order to fulfil the growing energy demand in the region (ANA 2002). Almost all the known subpopulations of *H. pumila* coincide with areas currently flooded, and might already have gone extinct, due to the construction of the aforementioned water dams. The few extant subpopulations vary from medium to large, with many clones and mature individuals. Nonetheless, they are currently strongly threatened due to pollution, deforestation, and by ongoing and future constructions of new hydropower plants. Thus, following the IUCN recommendations (IUCN 2001), *H. pumila* should be considered as Critically Endangered [CR, A2acd+B1b(ii, iii, iv)+B2ab(ii, iii, iv)+C1+E].

##### Morphological notes.

Extensive morpho-ecological studies (Horn 1983, 1988) have shown that *Heteranthera* species are highly polymorphic vegetatively, as an adaptation to submersion and variations in water level. The same can be observed in the new species herein described, that despite the diminute general stature, may sometimes possess extremely long petioles and peduncles. *Heteranthera pumila* has been kept in cultivation by the senior author, and even under different environmental conditions, little change was observed in the species’ vegetative morphology. Nevertheless, when cultivated in aquariums with different water depths, the change in the length of petioles and peduncles could be observed in less than a week. The already existing structures elongated in order to keep the leaf blades floating and flowers emerged, and the subsequently produced petiolate leaves and inflorescences were considerably longer than the previous ones of the same individual.

##### Affinities.

**Figure 5.**
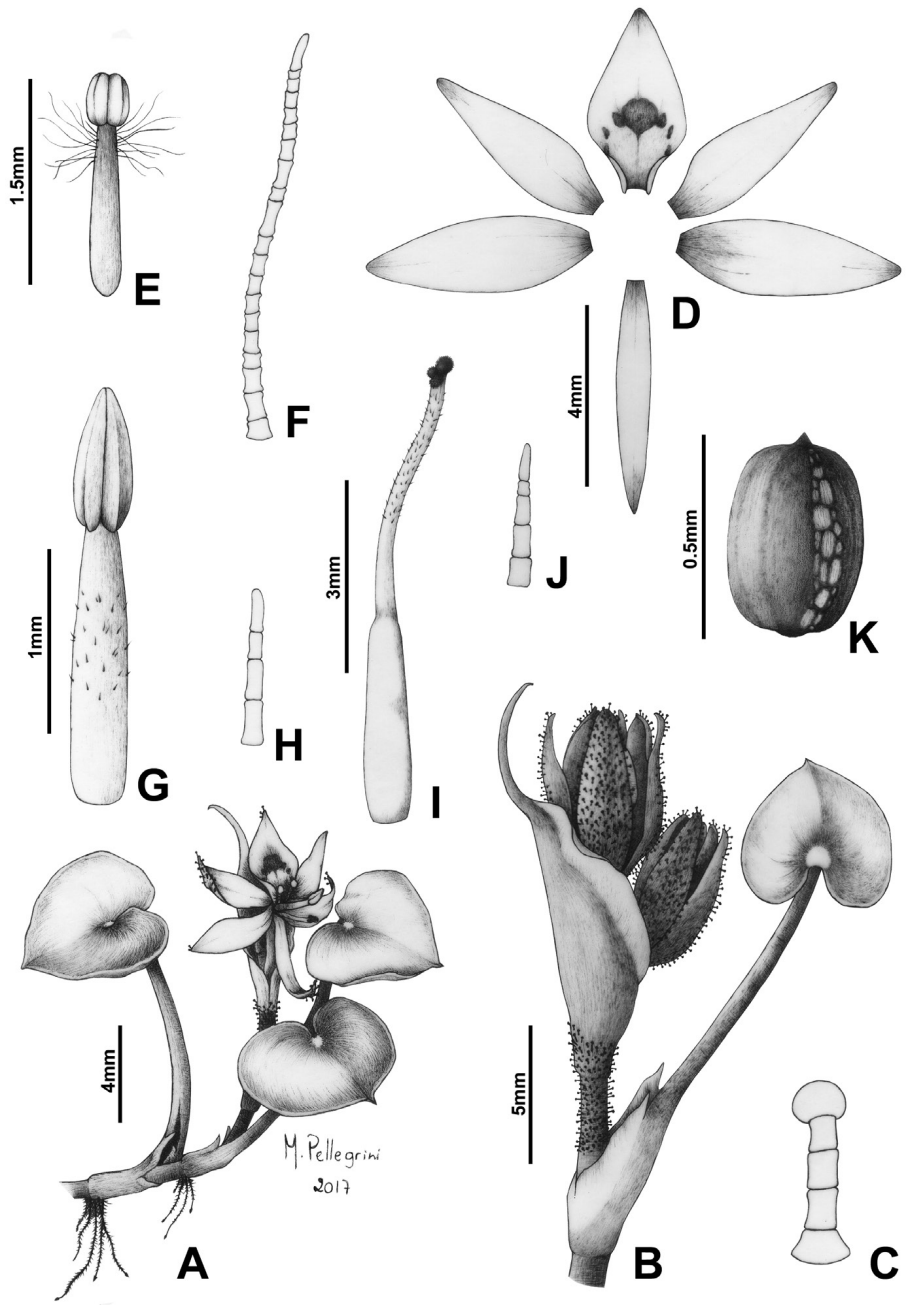
Line drawing of *Heteranthera pumila* M.Pell. & C.N.Horn. **A** Habit **B** Detail of the apex of the stem, showing a petiolate leaf, the ligule and a pre-anthesis 2-flowered inflorescence **C** Glandular hair from the inflorescence, perianth tube and lobes **D** Dissected perianth lobes, showing the 5+1 arrangement **E** Lateral stamen. **F** Uniseriate hair from the lateral stamen **G** Central stamen **H** Eglandular hair from the central stamen **I** Gynoecium, showing the stigma **J** Eglandular hair from the style **K** Detail of the inconspicuously winged seed, showing the persistent funiculus with raphid crystals. Illustration by M.O.O. Pellegrini, based on the holotype.

*Heteranthera pumila* is morphologically similar to *H. reniformis* due to its petiolate leaves with blades two or more times wider than long, cordate to reniform, rarely narrowly cordate, cincinnus enclosed by the basal bract, glandular-pubescent cincinnus axis, perianth lobes with a 5+1 arrangement with acute to acuminate apex, filaments straight, lateral stamens apically barbate, central stamen basally sparsely villose, and intrusive-parietal placentation (Horn 1985). It is also similar to *H. multiflora* due to its petiolate leaves with blades two or more times wider than long, cordate to broadly cordate to reniform, rarely narrowly cordate, perianth lobes with a 5+1 arrangement and acute to acuminate apex, and straight filaments (Horn 1985). Nonetheless, it can be easily differentiated from all remaining species of *Heteranthera* by its petiolate leaves with diminute blades [i.e. 3.5–11.8–(13.2) × 3.2–12.1 mm], inflorescences 1–2–(3)-flowered, peduncle densely glandular-pubescent, basal bract basally glandular-pubescent with aristate apex, and seeds smooth or with 7–9 inconspicuous longitudinal wings (Fig. 5). The only other species in *Heteranthera* that possesses seeds not conspicuously winged is *H. gardneri*, in which the wings are very short, giving the seeds a striate appearance. Nevertheless, in *H. pumila*, the testa is almost smooth, with the stripes representing only pigmentation. All the remaining species of *Heteranthera* possess seeds with 8–19 conspicuous longitudinal wings (Horn 1985; Table 1).

### Key to the species of *Heteranthera* in Brazil

**Table:** 

1	Ligule with several filiform (leaf-like) projections, sessile leaves appearing verticillate, blades filiform to acicular; flowers non-enantiostylic, stamen 1, staminodes commonly absent, if present consisting of a filiform projection	***H. gardneri* (Hook.f.) M.Pell.**
–	Ligule 2-parted, sessile leaves clearly distichously or spirally-alternate, blades linear oblong to narrowly obovate; flowers enantiostylic, stamens 3, staminodes generally absent, if present not filiform	**2**
2	Sessile leaves persistent in mature plants, petiolate leaves rarely produced, floating, blades linear oblong to narrowly obovate	**3**
–	Sessile leaves marcescent in mature plants, rarely persistent, petiolate leaves always produced, floating or emersed, blades narrowly cordate to broadly cordate to reniform or broadly ovate to broadly elliptic	**4**
3	Inflorescences 5–12-flowered, glandular-pubescent when emersed, basal bract (spathe) with aristate apex; perianth yellow, rarely lilac or white	***H. seubertiana* Solms**
–	Inflorescences (1–)2-flowered, always glabrous, basal bract (spathe) with mucronate to retuse apex, perianth lilac to purple	***H. zosterifolia* Mart.**
4	Petiolate leaves typically with blades longer than wide, base rounded to auriculate; perianth lobes with a 3+3 arrangement, nectar guide yellow to bright yellow, filaments sigmoid, glandular-pubescent, placentation axial	**5**
–	Petiolate leaves typically with blades wider than long, base conspicuously cordate; perianth lobes with a 5+1 arrangement, nectar guide yellowish green to green, filaments straight, barbate or villose with eglandular hairs, sometimes glabrous, placentation intrusive-parietal	**7**
5	Sessile leaves abaxially green; inflorescence (1–)2-flowered, spathe flattened, slightly to distinctly falcate, narrowly ovate to ovate, apex obtuse; perianth lobes obovate to broadly elliptic, three superior lobes without a white band at base	***H. oblongifolia* Mart. *ex* Schult. & Schult.f.**
–	Sessile leaves abaxially white; inflorescence 1-flowered, spathe cylindrical, straight, linear to narrowly obovate, apex acute to acuminate; perianth lobes oblong to linear elliptic, three superior lobes with a white band at base	**6**
6	Leaf blades rounded to oblong, cordate to truncate at base; floral tube glandular-pubescent, perianth lobes slightly to distinctively falcate, upper central perianth lobe auriculate near base; pollen dispersed in monads	***H. rotundifolia* (Kunth) Griseb.**
–	Leaf blades oblong to ovate, truncate to cuneate at base; floral tube glabrous, perianth lobes flat, upper central perianth lobe not auriculate; pollen dispersed in tetrads	***H. limosa* (Sw.) Willd.**
7	Petiolate leaves with smaller blades, 3.5–11.8–(13.2) × 3.2–12.1 mm; inflorescences 1–2–(3)-flowered, basal bract (spathe) with aristate apex; seeds smooth or with 7–9 inconspicuous longitudinal wings	***H. pumila* M.Pell. & C.N.Horn**
–	Petiolate leaves with larger blades, 12–75 × 10–81 mm; inflorescences 3–30-flowered, basal bract (spathe) with acute to mucronate, rarely spatulate-mucronate apex; seeds with 8–19 conspicuous wings	**8**
8	Petiolate leaves glandular-pubescent when emersed; inflorescence sessile, 10–30-flowered, flowers opening over several days, peduncle densely glandular-pubescent; central superior perianth lobe without a nectar guide, apex obtuse	***H. spicata* C.Presl**
–	Petiolate leaves always glabrous; inflorescence pedunculate, 3–8–(9–17)-flowered, flowers opening in one or two days, peduncle glabrous; central superior perianth lobe with a nectar guide, apex acute to acuminate	**9**
9	Inflorescences with flowers condensed at the base and apex of the cincinnus, 6–17-flowered, basal bract with spatulate-mucronate apex; perianth lobes externally glabrous, central superior perianth lobe 6.6–9.2 mm long; central stamen with filament glabrous, style glabrous	***H. catharinensis* C.N.Horn & M.Pell.**
–	Inflorescences with flowers evenly distributed on cincinnus, 3–13-flowered, basal bract with acute to mucronate apex; perianth lobes externally glandular-pubescent, central superior perianth lobe 2.3–5 mm long; central stamen with filament villose or barbate, style villose	**10**
10	Leaf blade cordate (length/width ~ 1); peduncle < 1 cm long, cincinnus main axis glabrous; all filaments barbate with long, purple hairs	***H. multiflora* (Griseb.) C.N.Horn**
–	Leaf blade commonly reniform (length/width mostly < 1); peduncle > 1 cm long, cincinnus main axis glandular-pubescent; lateral stamens barbate with long hairs, central stamen sparsely villose, hairs white	***H. reniformis* Ruiz & Pavón**

## Discussion

### Inflorescence morphology and terminology in Pontederiaceae

The inflorescence in Pontederiaceae, has traditionally been regarded as consisting of panicles and spikes, or more rarely, reduced to one-flowered racemose inflorescence (Lowden 1973; Dahlgren et al. 1985; Horn 1985; Rosatti 1987; Cook 1998). Nonetheless, some studies have described the inflorescence in the family as being thyrsoid, with an indeterminate main axis and cymose branches (Cook 1989; Richards and Barrett 1984; Pellegrini 2017). More specifically, Richards and Barrett (1984), based on developmental studies in *E. paniculata* (Spreng.) Solms, described the cymose secondary branches as representing cincinni with greatly reduced bracteoles. This is consistent with the commonly zig-zag or scorpioid pattern observed in many Pontederiaceae inflorescences (Pellegrini and Horn, pers. obs.), the occurrence of mirror-image flowers in *H. gardneri* (Hook.f.) M.Pell. (which is comparable to the 2-flowered cincinni with mirror-image flowers of Marantaceae; Kirchoff 1985), and the predominant occurrence of cincinni and other cymose inflorescences in Commelinid Monocots (Fahn 1953; Uhl 1969; Kirchoff 1985; Panigo et al. 2011; Kellogg et al. 2013; Remizowa et al. 2013; Stützel and Trovó 2013). Thus, the inflorescence in the family is to be regarded as thyrsoid, being composed of a many-branched thyrse, with spirally arranged cincinni in *Pontederia* *s.l.*, and reduced to a solitary cincinnus in *Heteranthera* *s.l.* Cincinni bracts and bracteoles are greatly reduced in most species, being not observable to the naked eye, but consisting of ephemeral rudimentary ridges under the scanning electron microscope (Richards and Barrett 1984). Bracteoles are only macroscopically visible in *E. meyeri* A.G.Schulz, a species closely related to *E. paniculata*, being a key character in differentiating both taxa (Horn 1998).

Inflorescence architecture, has a great unexplored taxonomic potential in the Pontederiaceae, also supporting the family’s bigeneric circumscription, proposed by Pellegrini (2017). Aside from that, different inflorescence patterns seem to support different lineages within the family’s two major clades. In *Heteranthera* *s.l.*, the reduction to 1–2-flowered inflorescence seems to be, at least, partially correlated with a reversal from intrusive-parietal placentation to axial placentation, and sigmoid filaments in the *H. limosa* (Sw.) Willd. species group [i.e. *H. limosa*, *H. lutea*, *H. oblongifolia* Mart. *ex* Schult. & Schult.f., and *H. rotundifolia* (Kunth) Griseb.]. Furthermore, in the permanently submersed species of *Heteranthera* [i.e. *H. dubia* (Jacq.) MacMill., *H. gardneri*, and *H. zosterifolia* Mart.], reduction to 1–2-flowered inflorescence seems to be correlated with the partial or complete loss of petiolate leaves, with the reversion from zygomorphic to actinomorphic flowers, and the loss of enantiostyly. In *Pontederia* *s.l.*, *E. meyeri* and *E. paniculata* can be readily differentiated from the remaining species on the clade by their elongated cincinni, and inflorescence erect at post-anthesis. In *Monochoria* C.Presl, the cincinni can range from obviously spirally arranged to fascicle-like, and from one to several-flowered, being very useful in species delimitation. Furthermore, great reduction is observed in the inflorescences of *E. diversifolia* (Vahl) Urb. and *E. natans* (P.Beauv.) Solms, with thyrsi always producing 1-flowered cincinni, and the number of cincinni being useful in differentiating both species. Finally, in *Pontederia* *s.s.*, the inflorescence is a spike-like thyrse, due to the increase in the number of cincinni, contraction of the cincinni peduncle and internodes, and finally, due to the shortening of the main florescence internodes.

### *Heteranthera reniformis* species complex and *H. multiflora* subcomplex

**Figure 6.**
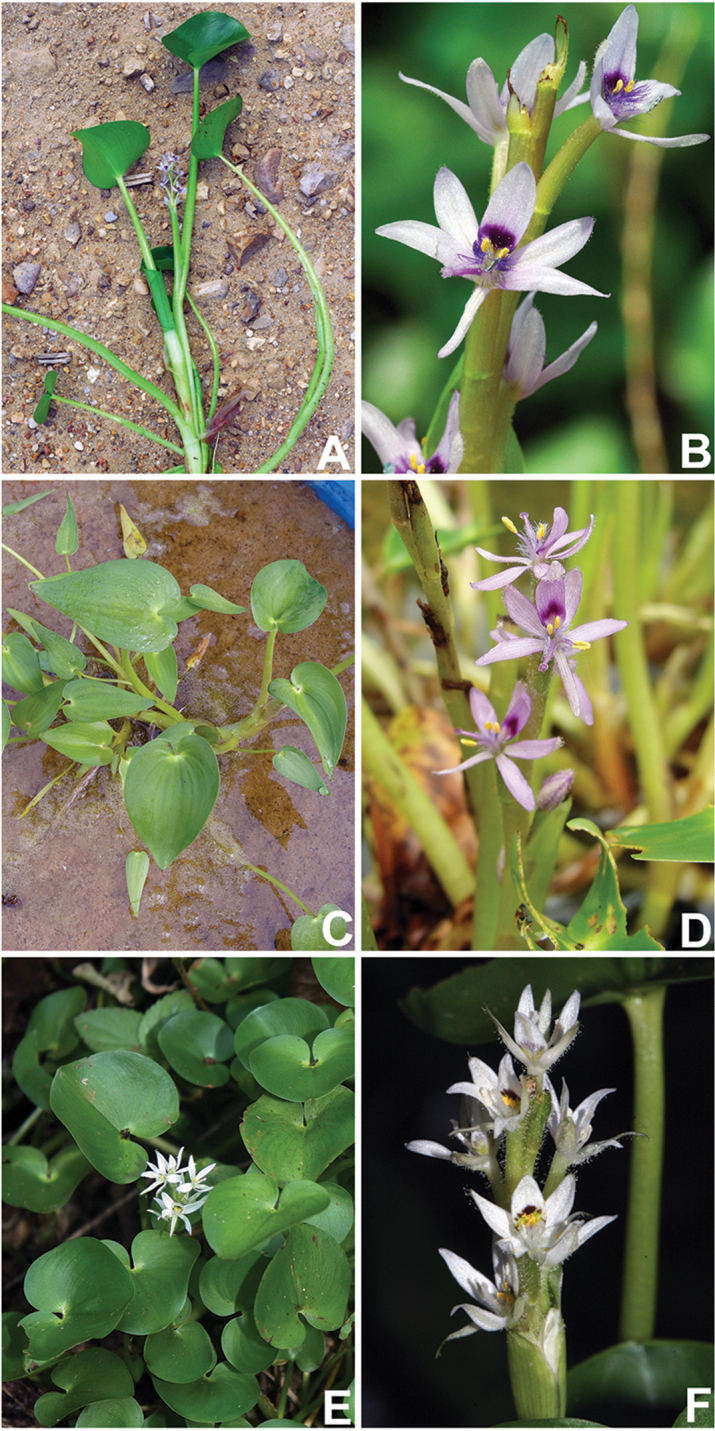
*Heteranthera reniformis* Ruiz & Pavón complex. **A–B** *H. multiflora* (Griseb.) C.N.Horn *s.l.*, from Missouri, USA: **A** Habit **B** Inflorescence **C–D** *H. peduncularis* Benth., from Michoacán, Mexico: **C** Habit **D** Inflorescence **E–F** *H. reniformis* Ruiz & Pavón *s.s.*, from Bahia state, Brazil: **E** Habit **F** Inflorescence. Photos A–B by Steve R. Turner, C–D by C.N. Horn, and E–F by M.O.O. Pellegrini

As aforementioned, *H. reniformis* *s.l.* is an economically important, but poorly understood weed. This species complex can be easily characterized by its petiolate leaves typically with blades wider than long, base conspicuously cordate; flowers opening in one or two days; perianth lobes with a 5+1 arrangement and acute to acuminate apex, nectar guide yellowish green to green; straight filaments, barbate or villose with eglandular hairs, sometimes glabrous; and intrusive-parietal placentation (Figs 1, 2, 4–6). The group is currently composed of five neotropical species: *H. catharinensis* (Figs 1, 2), *H. multiflora* *s.l.* (Fig. 6A, B), *H. peduncularis* (Fig. 6C, D), *H. pumila* (Figs 4, 5), and *H. reniformis* *s.s.* (Fig. 6E, F). Characters such as inflorescence architecture, pubescence, and flower morphology are key in species delimitation (Pellegrini and Horn, pers. obs.).

Despite our present contribution to the *H. reniformis* species complex, further studies are still necessary to better understand some polymorphic species. *Heteranthera multiflora* *s.l.* is widely but disjunctively distributed, occurring in the United States, Venezuela, and widespread across Brazil, Argentina, and Paraguay (Horn 1985). It is currently circumscribed as comprising plants with many-flowered inflorescences with most flowers exerted from the basal bract, glabrous cincinnus axis, and stamens bearded with long, uniseriate, purple hairs (Horn 1985; Horn 2002; Horn and Pellegrini, pers. obs.). However, throughout this species’ range, it is possible to recognize five different morphotypes: (1) specimens with petiolate leaf blades longer than wide, smaller sessile inflorescences, with most flowers included in the basal bract, flowers white to pale lilac, and distributed along the Atlantic Coast of the United States; (2) specimens with round petiolate leaf blades, longer sessile inflorescences, with few flowers included in the basal bract, flowers lilac to blue with darker perianth lobes base, and distributed in the Great Plains of the United States; (3) a sole peculiar collection from northern Venezuela; (4) specimens with petiolate leaf blades longer than wide, sessile inflorescences, lilac flowers, and distributed in Northeastern Brazil (i.e. states of Alagoas, Bahia, Paraíba, Pernambuco and Sergipe); and (5) specimens with petiolate leaf blades as wide as long, pedunculate inflorescences, white flowers, and distributed from Northern, Northeastern and Central-Eastern Brazil (i.e. states of Alagoas, Bahia, Maranhão, Mato Grosso do Sul, Pará, Rondônia, and Tocantins) to Southeastern Brazil (i.e. states of Espírito Santo, Minas Gerais, and Rio de Janeiro), Argentina, and Paraguay (Horn 1985; Horn and Pellegrini, pers. obs.). A new circumscription for *H. multiflora* *s.l.*, based on macromorphology and morphometric analyses, is currently in the works (Horn and Pellegrini, in prep.), and will shed new light in this poorly understood taxon.

## Supplementary Material

XML Treatment for Heteranthera catharinensis

XML Treatment for Heteranthera pumila
